# Analysis of risk factors and construction of a predictive model for severe acute pancreatitis complicated by sinistral portal hypertension

**DOI:** 10.3389/fphys.2025.1512144

**Published:** 2025-03-17

**Authors:** Mengbo Xiao, Yu An, Ying Di, Yunfeng Cui

**Affiliations:** ^1^ Graduate School, Tianjin Medical University, Tianjin, China; ^2^ Department of Hepatobiliary and Hepatobiliary Medicine Pancreatic Surgery, Department of Surgery, Tianjin Nankai Hospital, Tianjin, China; ^3^ Nankai Clinical School of Medicine, Tianjin Medical University, Tianjin, China

**Keywords:** severe acute pancreatitis, sinistral portal hypertension, portal venous system vascular lesions, independent risk factors, nomogram, prediction model

## Abstract

**Objective:**

Sinistral portal hypertension (SPH) is a common complication of severe acute pancreatitis (SAP). Patients with SPH often present asymptomatic, but are at risk of gastrointestinal bleeding and abdominal bleeding due to the presence of varices of the corresponding vessels, which are often fatal. However, there is no prediction model for SAP combined with SPH. This study aimed to identify the risk factors of SAP combined with SPH and to construct a relevant predictive model using independent risk factors.

**Materials and methods:**

The clinical data of 431 SAP patients were collected in this study. According to the presence or absence of SPH, the patients were divided into SPH group (n = 126) and non-SPH group (n = 305), and 431 patients were randomly assigned to the training set and validation set. Univariate logistics regression analysis was used to screen out the variables with significant differences, and then backward stepwise regression method was used for multivariate logistic regression analysis to determine the independent risk factors of SAP combined with SPH. Then a prediction model was constructed and represented by a nomogram, and the model was verified by internal validation. Receiver operating characteristic (ROC) curve and calibration curve were used to evaluate the predictive ability and accuracy of the model, and decision curve analysis (DCA) was used to evaluate the clinical value of the model.

**Results:**

Multivariate logistic regression analysis showed that male, MCTSI score, white blood cell count (WBC), and portal venous system vascular lesions (PVPSL) were independent risk factors for SAP complicated with SPH. The area under the working curve (AUC) of the clinical nomogram in the training set was 0.95 (95% CI: 0.92–0.97),and the P value of the Hosmer-Lemeshow test of the calibration curve was 0.969. The AUC in the validation set was 0.98 (95%CI: 0.96–1.00), and the P value of the Hosmer-Lemeshow test of the calibration curve was 0.963. The DCA in the training set and the validation set showed good clinical applicability of the model.

**Conclusion:**

Male, MCTSI score, WBC and PVPSL are independent risk factors for SAP complicated with SPH. The establishment of prediction model for SAP complicated with SPH is of great significance for the prevention and treatment of SPH in clinical practice.

## Introduction

Acute pancreatitis (AP) refers to a disease characterized by the activation of pancreatic enzymes due to various causes, followed by local or systemic inflammatory responses in the pancreas, with or without changes in the function of other organs (Harris et al., 2013). Approximately 20% of patients develop moderately severe acute pancreatitis (MSAP) or severe acute pancreatitis (SAP). It is accompanied by necrosis of the pancreas or peripancreatic tissue or organ failure, and the mortality rate is as high as 20%–40% (Boxhoorn et al., 2020; Shah et al., 2018). The clinical course of SAP is complex, which can lead to a variety of serious complications, including local complications, regional complications and systemic complications. The main local complications were fluid accumulation in the pancreas and surrounding tissues, necrosis and infection, pancreatic abscess and pseudocyst. Regional complications included vascular complications, intestinal complications and biliary complications. systemic complications were mainly systemic inflammatory response syndrome (SIRS) and multiple organ dysfunction syndrome (MODS).

Portal venous system complications (PVSC) is an important part of the regional complications of SAP, mainly including portal venous system vascular lesions (PVSVL), and sinistral portal hypertension (SPH). SPH, also known as left-sided portal hypertension, occurs due to conditions such as acute or chronic pancreatitis, pancreatic cysts, pancreatic necrosis, or pancreatic tumors. These conditions can lead to thrombosis, narrowing, occlusion, and blood flow obstruction in the portal venous system, resulting in impaired blood return from the spleen to the portal vein. This, in turn, causes manifestations of portal hypertension such as splenomegaly, hypersplenism, and the formation of collateral circulation.

Previous literature indicates that SPH was detected in 3.3% of AP patients and 12.5% of MASP patients (Xie et al., 2019). It has been reported that 4%–17% of SPH patients may experience gastrointestinal bleeding, and about 1.2%–14.5% of patients will die from it (Sakorafas et al., 2000; Butler et al., 2011). However, there are few studies on the risk factors of SAP complicated with SPH, and most of them are predicted by laboratory indicators, while the organ function, clinical scores, and imaging indicators at the time of onset are relatively ignored. Moreover, most of these studies were single-center retrospective studies and did not establish a good clinical prediction model. Therefore, it is of great significance to develop a prediction model for SAP complicated with SPH for early identification and intervention or effective treatment of SAP complicated with SPH in clinical practice for improving prognosis and reducing related medical costs.

In this study, we used the easily collected clinical laboratory indicators, imaging indicators and clinical scores to identify the independent risk factors of SAP combined with SPH by multivariate Logistic regression analysis and established a prediction model. The performance of the model was validated and evaluated.

## Materials and methods

### Selection of patients

**FIGURE 1:**
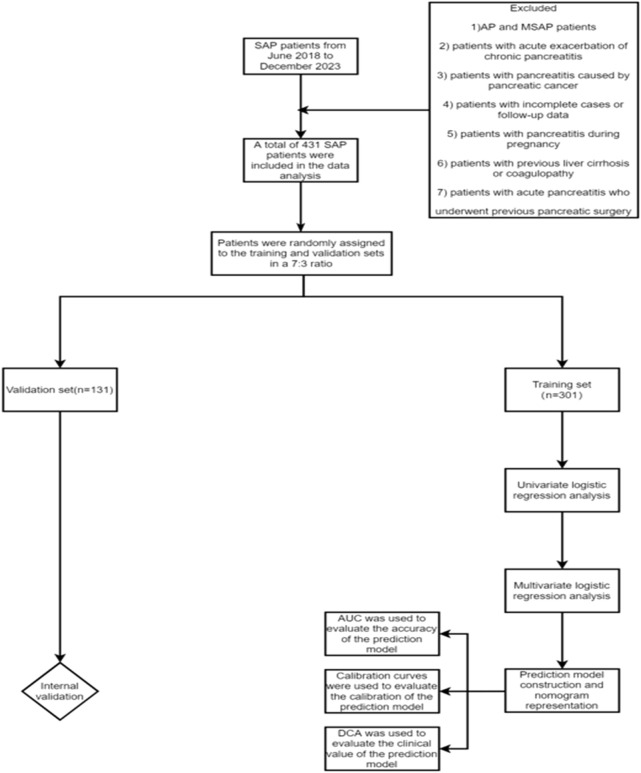
Study design flowchart of specific patient screening process.

This study retrospectively collected the clinical data of SAP patients admitted to Tianjin Nankai Hospital from June 2018 to December 2023. After screening based on inclusion and exclusion criteria, a total of 431 SAP patients were finally included. According to the presence or absence of SPH, the patients were divided into SPH group (n = 126) and non-SPH group (n = 305), and 431 patients were randomly assigned to the training set (n = 301) and validation set (n = 130). Inclusion criteria: 1) SAP patients who met the diagnostic criteria of the revised Atlanta classification in 2012; 2) patients with onset within 72 h; 3) age ≥18 years old; 4) complete clinical data. Exclusion criteria: 1) AP and MSAP patients; 2) patients with acute exacerbation of chronic pancreatitis; 3) patients with pancreatitis caused by pancreatic cancer; 4) patients with incomplete cases or follow-up data; 5) patients with pancreatitis during pregnancy; 6) patients with previous liver cirrhosis or coagulopathy; 7) patients with acute pancreatitis who underwent previous pancreatic surgery (Figure 1).

### Diagnostic criteria

SAP was defined according to the revised Atlanta classification (RAC) published in 2012: AP accompanied by persistent organ dysfunction (>48 h) (Banks et al., 2013).

PVSVL mainly include venous thrombosis, stenosis and occlusion. Thrombosis was defined as the filling defect without enhancement in the vascular lumen on the venous phase of enhanced CT. Stenosis was defined as the venous diameter of the lesion decreased by more than 50% compared with the upstream venous diameter without the filling defect in the vascular lumen on the venous phase of enhanced CT. Occlusion was defined as the complete absence of vascular imaging in the venous phase of enhanced CT (Easler et al., 2014).

Diagnostic criteria for SPH:Upper gastrointestinal radiography or endoscopy showed esophagogastric varices. Contrast-enhanced CT or magnetic resonance imaging of the portal vein system showed blood flow obstruction and collateral circulation in the portal vein system. Collateral circulation was defined as a disproportionate increase in caliber and number of vessels at any location in the portal system. The diagnostic criteria for common varicose veins due to SPH were a maximum diameter greater than 5 mm for the short gastric vein, 6 mm for the gastric coronary vein, 6 mm for the gastroepiploic vein, and 4 mm for the middle colonic vein. The rare abnormal varicose veins caused by sinistral portal hypertension, including pancreaticoduodenal vein and left adrenal vein, were judged by the changes of blood vessels during the course of the disease (Yu et al., 2022).

### Data collection

General characteristics: age, gender, height, weight, BMI.

Clinical data: history of smoking, history of alcohol consumption, history of diabetes, history of hypertension, history of abdominal surgery, previous pancreatitis, etiology.

Clinical scores: APACHE II score, Marshall score, MCTSI score, SOFA score, BISAP score, Rason score.

Diagnostics: SIRS, hepatic insufficiency, renal insufficiency, respiratory failure, CRRT, mechanical ventilation, sepsis, shock, necrotizing pancreatitis, degree of necrosis,SPH,PVSVL.

Laboratory tests: temperature, HCT, WBC, NEUT, LYC, MONO, PLT, Hb, CRP, LDL, D-dimer, APTT, PT, PTA, RDW, BUN, Fbg, NLR, PLR, SII, SIRI, K^+^, Na^+^, Ca^+^, Cr, UA, TP, ALB, TBIL, DBIL, GGT, ALP, ALT, TC, TG, PH, HCO3^-^, blood amylase, urine amylase.

### Statistical treatment

All statistical analyses were performed using R 4.4.0. The data of 431 patients were randomly divided into training set (301 cases) and validation set (130 cases) at a ratio of 7:3 using “caret” package for processing. The calculation of clinical characteristics was performed using the “compareGroups” package. For continuous variables, the normality test was performed first. All measurement data with normal distribution and homogeneity of variance were expressed as mean and standard deviation, and comparison between groups was analyzed by t-test. The measurement data with non-normal distribution or uneven variance were expressed as median (median) and quartile (IQR), and the Mann-Whitney U test was used for comparison. Categorical variables were expressed as counts and percentages and compared with the use of the chi-square test or Fisher’s exact test. The continuous variables with normal distribution were expressed as mean ± standard deviation, and those without normal distribution were expressed as median and interquartile range M(Q_1_, Q_3_). Categorical variables were presented as numbers and proportions. Differences between groups were assessed using the chi-square test, t-test, or Wilcoxon rank-sum test, depending on the data type. Logistic regression analysis was processed using the “rms” package. Firstly, univariate Logistic regression analysis was performed, and variables with P value less than 0.05 were included in backward stepwise multivariate Logistic regression analysis, and the screened independent risk factors were used to construct the model. The production of the Nomogram was processed with the “readr” package. Next, the models were tested for discrimination, calibration, and clinical applicability. The drawing of ROC curves and the calculation of AUC values were processed using the “pROC” package. Calibration curves were calculated and plotted using the “rms” package. Clinical decision curves were processed using the “rmda” package.

## Results

In the training set, patients were divided into the SPH group (n = 88) and the non-SPH group (n = 213) based on the occurrence of SPH. Through univariate logistic regression analysis (Table 1), it was found that gender, previous pancreatitis, SIRS, renal insufficiency, CRRT, mechanical ventilation, respiratory failure, sepsis, temperature, PVSVL, Marshall score, MCTSI score, SOFA score, WBC, NEUT, PT, RDW, and SIRI had a significant impact on the occurrence of SPH (*P* < 0.05). These variables were included in the multivariate Logistic regression analysis, and four independent risk factors for SAP complicated with SPH were finally screened by backward stepwise regression analysis (Table 2). Gender (*P* = 0.010), MCTSI score (*P* = 0.019), PVSVL (*P* < 0.001) and WBC(*P* = 0.010) were independent risk factors, and the clinical prediction model was constructed by these four independent risk factors.

**TABLE 1:** Univariate logistic regression results.

Variables	Univariate logistic analysis				
	β	S.E	Z	*P*	Or (95%CI)
Gender					
Female					1.00 (Reference)
Male	0.63	0.29	2.20	**0.028**	1.88 (1.07–3.30)
Smoking					
No					1.00 (Reference)
Yes	0.29	0.25	1.12	0.261	1.33 (0.81–2.19)
Alcohol consumption					
No					1.00 (Reference)
Yes	0.12	0.26	0.45	0.653	1.12 (0.68–1.86)
Abdominal surgery					
No					1.00 (Reference)
Yes	−0.09	0.30	−0.31	0.755	0.91 (0.51–1.64)
Diabetes					
No					1.00 (Reference)
Yes	−0.27	0.29	−0.92	0.355	0.76 (0.43–1.35)
Hypertension					
No					1.00 (Reference)
Yes	−0.06	0.27	−0.21	0.833	0.94 (0.55–1.61)
Previous pancreatitis					
No					1.00 (Reference)
Yes	0.52	0.27	1.96	**0.050**	1.68 (1.01–2.84)
Etiology					
HTG					1.00 (Reference)
Biliary	0.16	0.28	0.57	0.571	1.17 (0.67–2.04)
Alcoholic	0.22	1.23	0.18	0.861	1.24 (0.11–13.95)
After ERCP	−13.66	882.74	−0.02	0.988	0.00 (0.00 ∼ Inf)
Others	−0.88	1.09	−0.81	0.419	0.41 (0.05–3.51)
SIRS					
No					1.00 (Reference)
Yes	1.65	0.62	2.67	**0.008**	5.19 (1.55–17.41)
Hepatic insufficiency					
No					1.00 (Reference)
Yes	0.28	0.27	1.05	0.291	1.32 (0.79–2.23)
Renal insufficiency					
No					1.00 (Reference)
Yes	0.60	0.28	2.16	**0.031**	1.83 (1.06–3.16)
CRRT					
No					1.00 (Reference)
Yes	0.83	0.29	2.84	**0.005**	2.29 (1.29–4.07)
Mechanical ventilation					
No					1.00 (Reference)
Yes	0.73	0.29	2.53	**0.011**	2.08 (1.18–3.67)
Sepsis					
No					1.00 (Reference)
Yes	1.32	0.27	4.95	**<0.001**	3.73 (2.22–6.28)
Respiratory failure					
No					1.00 (Reference)
Yes	1.09	0.40	2.70	**0.007**	2.99 (1.35–6.61)
Shocks					
No					1.00 (Reference)
Yes	0.57	0.38	1.48	0.138	1.77 (0.83–3.76)
Necrotizing pancreatitis					
No					1.00 (Reference)
Yes	17.94	941.46	0.02	0.985	61,679,354.47 (0.00 ∼ Inf)
Degree of necrosis					
0					1.00 (Reference)
<30%	17.23	941.46	0.02	0.985	30,334,110.33 (0.00 ∼ Inf)
30%–50%	18.15	941.46	0.02	0.985	76,477,429.36 (0.00 ∼ Inf)
>50%	18.26	941.46	0.02	0.985	85,359,825.34 (0.00 ∼ Inf)
PVSVL					
No					1.00 (Reference)
Yes	5.01	0.62	8.12	**<0.001**	149.17 (44.56–499.29)
Age (year)	−0.01	0.01	−1.42	0.156	0.99 (0.97–1.01)
Height(m)	2.91	1.55	1.88	0.060	18.39 (0.89–380.79)
Weight (Kg)	0.01	0.01	0.59	0.554	1.01 (0.99–1.02)
BMI(Kg/m^2^)	−0.02	0.03	−0.48	0.629	0.98 (0.92–1.05)
Temperature (°C)	0.41	0.18	2.26	**0.024**	1.51 (1.06–2.17)
APACHE II	0.04	0.02	1.57	0.116	1.04 (0.99–1.08)
Marshall	0.21	0.09	2.19	**0.028**	1.23 (1.02–1.48)
MCTSI	0.38	0.07	5.25	**<0.001**	1.46 (1.27–1.67)
SOFA	0.18	0.06	2.83	**0.005**	1.19 (1.06–1.35)
BISAP	0.11	0.17	0.64	0.520	1.11 (0.80–1.54)
Rason	0.15	0.08	1.77	0.077	1.16 (0.98–1.36)
HCT (%)	−0.00	0.01	−0.07	0.945	1.00 (0.97–1.03)
WBC(*10^9^/L)	0.09	0.02	3.68	**<0.001**	1.09 (1.04–1.15)
NEUT(*10^9^/L)	0.06	0.02	2.34	**0.019**	1.06 (1.01–1.11)
LYC (10^9^/L)	−0.03	0.19	−0.16	0.873	0.97 (0.67–1.41)
MONO (*10^9^/L)	0.63	0.36	1.76	0.079	1.88 (0.93–3.81)
PLT(*10^9^/L)	−0.00	0.00	−0.74	0.457	1.00 (1.00–1.00)
Hb(g/L)	−0.00	0.00	−0.81	0.419	1.00 (0.99–1.00)
CRP(mg/L)	0.00	0.00	1.30	0.195	1.00 (1.00–1.00)
LDL(mmol/L)	−0.04	0.09	−0.51	0.610	0.96 (0.81–1.13)
D-dimer (mg/L)	0.01	0.02	0.32	0.747	1.01 (0.97–1.05)
APTT(s)	0.02	0.02	1.42	0.157	1.02 (0.99–1.06)
PT(s)	0.20	0.09	2.15	**0.031**	1.22 (1.02–1.47)
PTA (%)	−0.01	0.01	−1.69	0.091	0.99 (0.97–1.00)
RDW (%)	0.06	0.03	1.99	**0.047**	1.06 (1.01–1.13)
BUN(mmol/L)	−0.01	0.04	−0.29	0.769	0.99 (0.90–1.08)
Fbg(g/L)	0.01	0.02	0.24	0.810	1.01 (0.96–1.06)
NLR	0.01	0.01	1.31	0.191	1.01 (0.99–1.04)
PLR	−0.00	0.00	−0.03	0.977	1.00 (1.00–1.00)
SII	0.00	0.00	1.53	0.127	1.00 (1.00–1.00)
SIRI	0.04	0.02	2.14	**0.032**	1.04 (1.01–1.07)
K+(mmol/L)	0.29	0.17	1.65	0.098	1.33 (0.95–1.88)
Na+(mmol/L)	−0.00	0.01	−0.43	0.666	1.00 (0.99–1.01)
Ca+(mmol/L)	0.00	0.00	0.57	0.572	1.00 (1.00–1.00)
Cr (umol/L)	0.00	0.00	0.70	0.481	1.00 (1.00–1.00)
UA (umol/L)	0.00	0.01	0.17	0.861	1.00 (0.98–1.03)
TP (g/L)	−0.00	0.02	−0.15	0.880	1.00 (0.96–1.04)
ALB (g/L)	0.21	0.30	0.69	0.490	1.23 (0.68–2.24)
TBIL (mmol/L)	0.00	0.00	0.90	0.370	1.00 (1.00–1.01)
DBIL (mmol/L)	0.00	0.00	0.72	0.473	1.00 (0.99–1.01)
GGT(U/L)	0.00	0.00	0.53	0.596	1.00 (1.00–1.00)
ALP(U/L)	0.00	0.00	0.35	0.729	1.00 (1.00–1.00)
ALT(U/L)	−0.01	0.03	−0.39	0.700	0.99 (0.93–1.05)
TC(mmol/L)	0.00	0.00	0.05	0.961	1.00 (1.00–1.00)
TG(mmol/L)	−0.00	0.01	−0.20	0.845	1.00 (0.99–1.01)
PH	1.81	1.87	0.97	0.333	6.11 (0.16–239.21)
HCO3-(mmol/L)	−0.01	0.03	−0.30	0.766	0.99 (0.94–1.05)
Blood amylase (U/L)	0.00	0.00	0.14	0.892	1.00 (1.00–1.00)
Urine amylase (U/L)	0.00	0.00	0.43	0.670	1.00 (1.00–1.00)

**TABLE 2:** Multivariate logistic regression results.

Variables	*P*	OR (95%CI)
Gender	0.010	3.24 (1.32 ∼ 7.96)
MCTSI	0.019	1.35 (1.05 ∼ 1.73)
WBC	0.010	1.11 (1.03 ∼ 1.20)
PVSVL	<0.001	150.00 (42.17 ∼ 533.53)

### Construction of a nomogram

**FIGURE 2:**
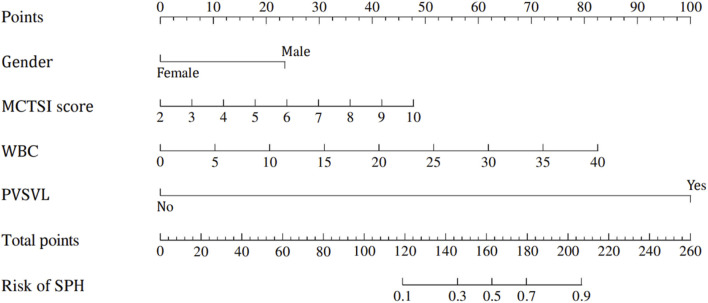
Nomogram for prediction model of severe acute pancreatitis complicated with sinistral portal hypertension.

According to the results of multivariate Logistic regression, a nomogram was drawn based on the four independent risk factors of SAP combined with SPH. Different clinical conditions corresponded to different scores, and then the scores of each variable were added together. Finally, a line was drawn on the total score line corresponding to the risk factor axis to predict the risk of SPH in SAP patients. (Figure 2).

### Performance verification of the nomogram in the training and validation sets

**FIGURE 3:**
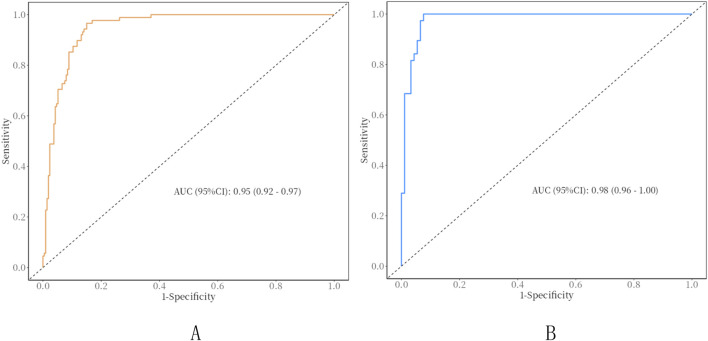
ROC curves of clinical nomograms. **(A)** Training set; **(B)** validation set; AUC, Area under the curve.

**FIGURE 4:**
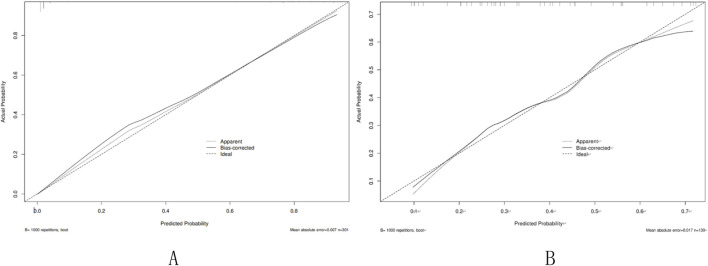
Calibration curve of the nomogram. **(A)** Training set; **(B)** Validation set. Calibration curve of the nomogram. The X-axis represented the predicted possible SPH risk. The Y-axis represented the actual diagnosed SPH. The diagonal dotted line meant a perfect prediction by an ideal model. The short-dashed line represented the apparent prediction of nomogram, and the solid line was bias-corrected by bootstrapping (B = 1,000 repetitions), indicating observed nomogram performance.

**FIGURE 5:**
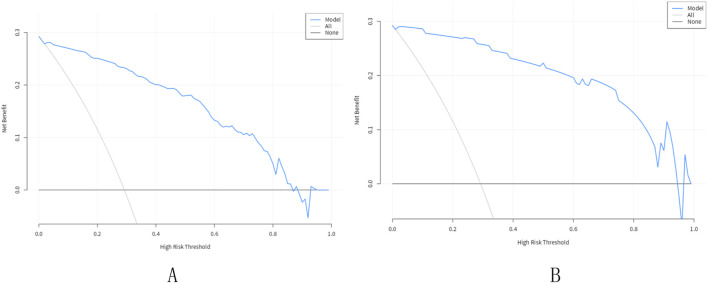
Decision curve analysis for the nomogram. **(A)** Training set; **(B)** Validation set. The X-axis showed the threshold probability. The Y-axis measured the net benefit. The blue solid line represented the nomogram. The gray solid line represented the assumption that all subjects were Pancreatic sinistral portal hypertension.

In the training set, In the training set, the AUC of the nomogram is 0.95 (95% CI: 0.92–0.97), with a cut-off value of 0.308, a sensitivity of 0.85 (95% CI: 0.80–0.90),a specificity of 0.97 (95% CI: 0.93–1.00), and a accuracy of 0.88 (95% CI: 0.84–0.92), a PPV of 0.91 (95% CI: 0.78–0.98), a NPV of 0.94 (95% CI: 0.92–0.98). In the Validation set, the AUC of the nomogram is 0.98 (95% CI: 0.96–1.00), with a cut-off value of 0.308,a sensitivity of 0.92 (95% CI: 0.87–0.98), a specificity of 0.97 (95% CI: 0.92–1.00), and a accuracy of 0.94 (95% CI: 0.80–0.97),a PPV of 0.91 (95% CI: 0.88–1.00), a NPV of 0.97 (95% CI: 0.97–0.99) (Figure 3). The P value of Hosmer-Lemeshow test of calibration curve in the training set was 0.969. The Bootstrap method was used for internal validation with 1000 replicates and the calibration curve was drawn. The *P* value of the Hosmer-Lemeshow test of the calibration curve in the training set was 0.969. The *P* value of the Hosmer-Lemeshow test for the calibration curve in the validation set was 0.963 (Figure 4). The DCA curves were used to reflect the net benefits of the prediction model (Figure 5). In general, the prediction model showed good consistency and good predictive ability in the training and validation sets.

## Discussion

SAP can lead to thrombus formation within the vessels of the portal venous system, or result in vascular narrowing, occlusion, and blood reflux disorders. This vascular pathology can obstruct the pathway of blood returning from the spleen to the portal vein, leading to increased venous pressure in the upstream splenic vein and collateral vessels. This can cause secondary splenomegaly and congestion, and even result in hypersplenism, as well as the development of esophageal varices between the spleen and stomach, referred to as SPH (Li et al., 2019; Ito et al., 2008). At present, there are few studies on the risk factors of SAP complicated with SPH, and no good clinical prediction model has been established. In this study, we concluded that male, increased MCTSI score, PVSVL, and increased WBC were independent risk factors for SAP complicated with SPH, and a clinical prediction model was established by these indicators. Through internal validation, the prediction model showed good predictive ability and clinical practical value. Therefore, the study of risk factors and the establishment of prediction model in this study are of great value for clinically predicting the risk of SPH in SAP patients and improving their prognosis.

The formation of SPH is closely related to the patency of blood vessels in the portal vein system. The splenic vein is the most common abnormal vein, followed by the portal vein and the superior mesenteric vein. The previous definition of SPH is regional portal hypertension caused by splenic vein thrombosis (Madsen et al., 1986),With the progress of research, Yu et al. showed that splenic vein stenosis and occlusion were independent risk factors for MSAP complicated with SPH (Yu et al., 2022), Zhao et al. showed that splenic vein stenosis or occlusion is an independent risk factor for AP complicated with SPH formation (Zhao et al., 2023), In addition, some Chinese studies have shown that not only splenic vein thrombosis can lead to SPH, but also the lesions (stenosis, occlusion, thrombosis) of the splenic vein can lead to SPH. In addition, some Chinese studies have shown that not only splenic vein thrombosis can lead to SPH, but also the lesions (stenosis, occlusion, thrombosis) of the splenic vein may lead to SPH (Guoyu et al., 2023; Zheng et al., 2023). In this study, PVSVL (*P* < 0.001) is an independent risk factor for SPH in SAP, and the incidence of portal vein system stenosis is significantly higher than the incidence of thrombosis, which indicates that in addition to thrombosis, the factors of portal vein system invasion may include stimulation of pancreatic enzymes and inflammatory mediators, coagulation dysfunction, pressure of necrotic tissue, etc. This is consistent with previous studies, and also proves that vascular lesions in the portal vein and superior mesenteric vein are also likely to cause SPH, which has also been proved by previous case reports (Duan et al., 2015; Wan-Li et al., 2022).

Male was an independent risk factor for SAP complicated with SPH in this study, which is consistent with previous studies (Li et al., 2019; Xie et al., 2019; Yu et al., 2022). In addition, in some studies, male is a high risk factor for SVT in AP patients (Toqué et al., 2015). Therefore, we speculate that men are more likely to develop portal vein thrombosis than women in SAP patients, which may lead to a higher risk of SAP complicated with SPH. In addition, the existing literature has not analyzed the pathophysiological reasons for this result from the perspective of genetics, and this risk factor needs to be further studied (Roach et al., 2014).

The MCTSI is a clinical radiological imaging system, which is mainly used to assess the degree of peripancreatic fluid accumulation and pancreatic necrosis in acute pancreatitis (AP). In previous studies, it has also been used as a risk factor to predict pancreatic complications and participate in the construction of prediction models. In this study, high MCTSI score is an independent risk factor for SPH in SAP, which is also consistent withthe results of previous studies (Zhao et al., 2023; Guo et al., 2024). But the MCTSI is highly subjective, and different radiologists may give different scores; Therefore, a unified scoring method for the severity of pancreatitis is necessary and needs to be studied.

In the present study, elevated WBC was an independent risk factor for SPH in SAP, which was also consistent with previous studies (Guo et al., 2024). In addition, WBC is also an independent risk factor for gastrointestinal bleeding caused by gastric varices rupture in SPH patients (Wang, 2022). Therefore, it is of great significance to include WBC in the establishment of prediction model. We hypothesized that because WBC is an important indicator of the severity of pancreatitis and the degree of infection and necrosis, the increase of WBC may indicate the aggravation of local pancreatic inflammation and the appearance of infection and necrosis, which are considered to be related to the occurrence of SPH (Chang-kun et al., 2022; Wang et al., 2022).

This study has certain limitations. 1) This study is a single-center retrospective study, and there is a certain information bias and selection bias. During the follow-up period, a certain number of patients may be missed, which reduces the estimated incidence of SPH and may have a certain impact on the screening of risk factors and the construction of prediction models. Therefore, prospective studies and the establishment and validation of prediction models are needed to improve the results. 2) The establishment and validation of the prediction model in this study were based on the data of a single center, and there was a lack of validation of the model by external medical centers. Therefore, large sample data and multi-regional and multi-center data need to be further tested and updated. 3) Since SPH may cause serious complications such as abdominal bleeding and gastrointestinal bleeding, our model was only able to predict the risk of SPH, but not the complications of SPH, which can be the focus of our next research.

## Conclusion

Male, elevated WBC, elevated MCTSI score, and PVSVL are independent risk factors for SAP complicated with SPH. A clinical prediction model for SAP complicated with SPH was developed and validated, and a nomogram was drawn to help clinicians better identify the risk degree of SAP complicated with SPH.

### Limitation

This study has certain limitations. 1) This study is a single-center retrospective study, which is subject to information bias and selection bias. During the follow-up period, a certain number of patients may be missed, which reduces the estimated incidence of SPH and may have a certain impact on the screening of risk factors and the construction of prediction models. Therefore, prospective studies and the establishment and validation of prediction models are needed to improve the results. 2) The establishment and validation of the prediction model in this study were based on the data of a single center, and there was a lack of validation of the model by external medical centers. Therefore, large sample data and multi-regional and multi-center data need to be further tested and updated. 3) Since SPH may cause serious complications such as abdominal bleeding and gastrointestinal bleeding, our model was only able to predict the risk of SPH, but not the complications of SPH, which can be the focus of our next research. 4) Due to the unequal sample size between SPH and non-SPH groups in this study. This biased the values of effectiveness indicators, and SMOTE method should be applied, but the authors did not apply it due to the shortcomings of statistical methods.

## Data Availability

The raw data supporting the conclusions of this article will be made available by the authors, without undue reservation.
